# Toward a human-centered digital ecosystem: bridging the “AI Chasm” in intelligent nosocomial infection monitoring

**DOI:** 10.3389/fpubh.2026.1879491

**Published:** 2026-07-08

**Authors:** Zufang Mou, Lina Diao, Weili Xu, Xiuxiang Luan

**Affiliations:** 1 Yantaishan Hospital, Yantai, China; 2 Woman and Children’s Hospital, Qingdao University, Qingdao, China

**Keywords:** artificial intelligence, clinical application, healthcare-associated infections, infection monitoring, intelligent nosocomial, internet of things, precision infection control

## Abstract

Healthcare-associated infections (HAIs) pose a persistent global threat to patient safety, rendering traditional, resource-intensive manual surveillance increasingly unsustainable. While intelligent nosocomial infection monitoring systems (INIMS) offer a transformative digital solution, their clinical integration is frequently derailed by the “AI Chasm”—a critical misalignment between algorithmic design and clinical ergonomics. This Perspective transcends theoretical performance metrics to dissect real-world socio-technical barriers, including alert fatigue, dataset shift, and medico-legal liability. Unlike traditional socio-technical models (such as the SEIPS or NASSS frameworks) that primarily focus on the retrospective evaluation of health IT usability, our framework provides a proactive, pre-deployment governance strategy. To bridge this implementation gap, we propose a human-centered governance ecosystem anchored in two practical frameworks: “silent-mode validation” for safe, prospective clinical debugging, and “data solidarity” to ensure equitable resource allocation, particularly in low- and middle-income countries (LMICs). Ultimately, this framework outlines an actionable pathway to translate isolated technological innovations into sustainable, clinically integrated public health tools.

## 1 Introduction

### 1.1 Background and significance

Driven by aging populations and rising multidrug-resistant organisms (MDROs), healthcare-associated infections (HAIs) continue to severely compromise global patient safety (1–4). Traditional infection prevention and control (IPC) frameworks are increasingly ill-equipped to handle these dynamic clinical risks. Relying predominantly on retrospective, resource-intensive manual medical record reviews, traditional IPC is inherently susceptible to sampling bias and the Hawthorne effect, which often inflates reported compliance rates while masking short-term transmission risks (5, 6).

The integration of digital health technologies, namely Big Data, Artificial Intelligence (AI), and the Internet of Things (IoT)—offers a transformative paradigm shift from retrospective reporting to real-time, proactive intelligent monitoring. However, the large-scale clinical adoption of Intelligent Nosocomial Infection Monitoring Systems (INIMS) is obstructed by a prevalent “AI Chasm.” This socio-technical implementation gap explains why machine learning models with exceptional laboratory metrics frequently fail to improve real-world treatment outcomes, plagued by fragmented cross-system integration, algorithmic opacity, and alert fatigue. Effective digital transformation requires prioritizing clinical ergonomics; therefore, this Perspective dissects these socio-technical barriers and proposes a human-centered, equity-oriented governance framework to facilitate the safe and sustainable implementation of INIMS.

### 1.2 Current research status

Globally, the implementation of intelligent infection monitoring is plagued by a prominent “digital paradox.” While high-income regions rapidly standardize automated early warning systems, low- and middle-income countries (LMICs)—which bear an exceptionally heavy endemic HAI burden, with ICU infection densities often triple those of the United States—remain structurally underserved. Currently, only 12% of machine learning clinical decision support systems (ML-CDSS) are adapted for LMICs, with virtually none designed for primary care settings where data extraction is notoriously challenging. Over-reliance on proprietary databases from high-income countries not only limits algorithmic generalizability but risks exacerbating global health inequities, as these automated protocols fail to align with resource-limited clinical realities. Even in well-resourced environments, clinical translation is frequently stalled by fragmented data integration and fundamental mismatches with clinical workflows (7–9).

**Figure 1:**
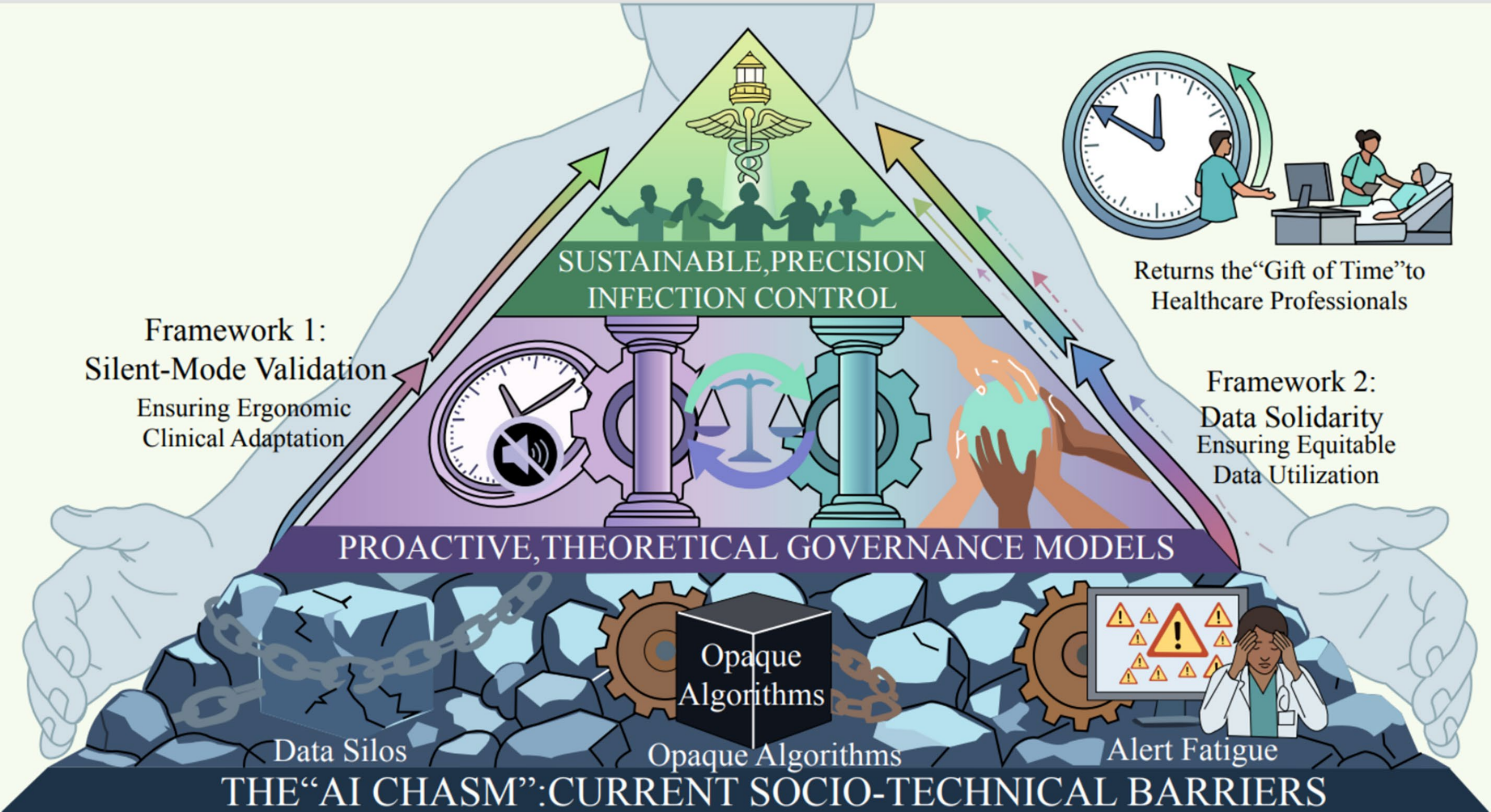
Bridging the “AI Chasm” through a human-centered digital ecosystem. This conceptual framework depicts the transition from existing socio-technical barriers, including data silos, algorithmic opacity, and alert fatigue, toward a sustainable model of precision infection control. Through the integration of silent-mode validation to facilitate ergonomically aligned clinical implementation and data solidarity to promote equitable use of healthcare data, the proposed ecosystem supports a proactive governance approach and helps restore protected clinical time for healthcare professionals.

While most existing literature focuses heavily on the technical nuances of algorithms, this Perspective distinguishes itself by defining the “AI Chasm” not as a mere technical deficit, but as a critical misalignment between algorithmic design and clinical ergonomics. To bridge this socio-technical gap, we propose a human-centered governance ecosystem anchored by two actionable frameworks anchored by two actionable frameworks (Figure 1):

1. Silent-Mode Validation: A prospective strategy allowing expert teams to debug real-time predictions in live clinical settings, preventing uncalibrated alerts from reaching frontline staff and mitigating alert fatigue.
2. Data Solidarity: A governance framework ensuring that the predictive value derived from aggregated clinical data is equitably returned to benefit all patient populations, particularly those in resource-constrained settings.

The justification for these two frameworks is directly derived from the inherent shortcomings of existing health IT implementation models and regulatory theories.

Regarding clinical integration, the assumption that algorithms can fully automate complex processes without prospective clinical oversight often neglects the operational fragility introduced by dataset shift (10). AI systems are not capable of fully replicating the contextual nuances of clinical practice; thus, human expertise is needed to rectify mistakes and ensure the responsible use of machine learning (11, 12). Therefore, ‘silent-mode validation’ is not just a technical trial phase but an essential theoretical requirement to safeguard human agency and prevent algorithmic errors from causing clinical harm prior to patient exposure.

Simultaneously, in the field of data governance, existing legislative frameworks primarily focus on increasing individual control over data. This individualistic privacy paradigm is increasingly inadequate to address the major power asymmetries between technology developers and healthcare institutions in the digital era. Hence, it is imperative to ground INIMS deployment in the concept of ‘Data Solidarity’. Through the prism of data solidarity, the predictive benefits derived from mass clinical data must be governed as a shared public good rather than a commercial commodity to be exploited. This ethical shift ensures the fair distribution of digital IPC benefits across healthcare systems worldwide, particularly in resource-poor LMICs (13–15).

### 1.3 Literature search strategy and selection criteria

To minimize selection bias while preserving the conceptual agility of a Perspective article, we bypassed rigid systematic review protocols (e.g., PRISMA) in favor of a purposive sampling strategy. We conducted a narrative literature search across PubMed, Scopus, and Web of Science using combinations of terms such as “intelligent nosocomial infection monitoring,” “artificial intelligence,” and “implementation science.”

Cited studies—particularly the real-world deployments summarized in Table 1—were selected based on three strict criteria: (1) empirical evaluation in real-world clinical settings (explicitly excluding purely theoretical or laboratory models); (2) concrete socio-technical implementation outcomes (e.g., workflow efficiency, interoperability); and (3) geographical diversity, ensuring representation across both high-income tertiary centers and resource-constrained LMICs. This robust selection methodology helps ensure our proposed framework remains deeply grounded in practical global realities.

**Table 1:** Summary of clinical application scenarios, predictive metrics, and implementation outcomes of INIMS.

Clinical application scenario	Study context and resource setting	Implementation scale/sample size	Ai chasm barrier addressed	Study design	Predictive performance metrics	Implementation and clinical outcomes	Ref.
3.1 Real-time automated surveillance	Iran (INIS) [LMIC]	1,066 hospitals(>11 million admissions)	Breaking data silos and standardization	National database analysis	N/A (data aggregation)	Unified national HAI diagnostic criteria; accurately mapped extreme MDRO resistance profiles.	(26)
	Europe (SmICS) [HIC]	Multicenter	Overcoming workload paradox	Workflow evaluation	N/A (workflow analysis)	Reduced routine IPC task time by up to 81.47%; saved an average of 39.2 min/routine.	(16)
	Brazil (MLP and RF) [LMIC]	>120,000 patient-days	Workflow automation and manual bottlenecks	Retrospective cohort	AUROC: 90.27%; Sensitivity: 88.57%	Significantly automated hospital-wide surveillance while minimizing manual chart review time.	(10)
3.2 Early warning and active intervention	USA (ICU) [HIC tertiary]	Multicenter icu	Mitigating alert fatigue	Retrospective cohort	AUROC: 0.87 (vs. standard logistic 0.79)	Integrated into EMR as a real-time dashboard; enabled IP prioritization of daily interventions.	(11)
	China (AI warning) [emerging /tertiary]	Single-center ICU	Bridging the diagnostic lag	Retrospective implementation	Pre-warning: 100%*; Recognition: 100%*	Contributed to a 45% decrease in overall ICU HAI incidence; median lead time of 85.2 days.	(14)
	Italy (SVM) [HIC]	Multicenter ICU	Enhancing cognitive depth	Multicenter retrospective	AUROC: 0.90; Accuracy: 88%	Surpassed traditional severity scores; enabled targeted day-one clinical interventions.	(22)
3.3 MDROs full-process management	Germany (Charité CLAR) [HIC tertiary]	1,714 clusters (hospital-wide)	Eliminating surveillance blind spots	Prospective implementation	High sensitivity in early detection	Detected 1,714 clusters; 79% involved pathogens previously missed by manual surveillance.	(13)
	China (MDRO system) [emerging/tertiary]	Single-center oncology	Spatio-temporal Interoperability	Single-center implementation	Identification rate: 100%*	Saved 4.71 h/day in IPC tasks; MRSA transmission dropped by 56%.	(14)
3.4 Refined IPC quality management	Global (IoT hand hygiene) [mixed]	Multiple validation studies	Overcoming hawthorne effect	Systematic review	N/A (Intervention Adherence)	Eliminated Hawthorne effect; improved short-term compliance with performance feedback.	(6)
	UK (ATP monitoring) [HIC]	Hospital-wide	Objective data quantification	Prospective surveillance	N/A (environmental metric)	Transitioned cleanliness assessment to objective data; significantly reduced VRE prevalence.	(4)
3.5 Publichealth emergency support	Global (COVID-19) [global]	Macro-level tracing	Establishing digital presence	Epidemiological modeling	N/A (contact tracing efficacy)	Successfully isolated carriers and reduced transmission delay to instantaneous alerts.	(19)
	China (DRL forecasting) [emerging/tertiary]	Multicenter	Dynamic resource optimization	Predictive modeling	N/A (simulation optimization)	Optimized non-pharmacological interventions; contained outbreaks within capacity limits.	(9)

The metrics and outcomes summarized in this table are drawn from highly heterogeneous study designs (ranging from single-center retrospective implementations to macro-level epidemiological modeling) and diverse resource settings. Consequently, the predictive performance metrics and implementation outcomes are not directly comparable. Furthermore, absolute performance claims (e.g., “100% pre-warning” or “100% recognition”) reported in certain single-center studies should be interpreted with extreme caution. These metrics often reflect highly controlled datasets and carry a significant risk of algorithmic overfitting; they are unlikely to generalize to broader, dynamic clinical environments without rigorous prospective validation.

## 2 Core technical support of INIMS

The efficacy of INIMS is anchored in a tripartite socio-technical architecture. First, IoT sensors facilitate continuous, objective data collection, transitioning cleanliness and exposure assessments away from subjective human observation. Second, standardized semantic interoperability breaks down traditional data silos, smoothly fusing inputs from hospital information systems (HIS), laboratory networks (LIS), and electronic medical records (EMR) to construct a holistic patient trajectory. Finally, algorithm-based risk stratification leverages this multi-source data fusion. By utilizing advanced machine learning (ML) and Natural Language Processing (NLP), INIMS can go beyond linear statistical assumptions to identify complex, non-linear infection patterns within both structured data and unstructured clinical text. Compared to conventional rule-based protocols, this integrated algorithmic approach significantly enhances predictive sensitivity and reduces redundant false alarms in dynamic clinical environments (16, 17).

## 3 Clinical core applications of INIMS

As INIMS transition from theoretical frameworks to clinical deployment, their real-world utility spans diverse medical scenarios. Automated data extraction, machine learning (ML) prediction models, and Internet of Things (IoT) sensors have demonstrably improved infection prevention and control (IPC) metrics. Table 1 summarizes the quantitative outcomes and specific study designs across five key application scenarios. While these metrics—ranging from reduced false negatives to decreased HAI incidence—are drawn from heterogeneous methodological settings and are not directly comparable, they highlight distinct regional priorities. For instance, European frameworks (e.g., SmICS) emphasize ergonomic integration and contact-tracing automation, whereas emerging healthcare systems leverage INIMS primarily to bridge critical data gaps, achieving near-complete recognition rates even in resource-constrained environments.

### 3.1 Real-time automated surveillance and precision reporting

INIMS can reduce the systematic blind spots and inherent reporting lags of fragmented manual chart reviews by continuously extracting high-fidelity clinical indicators and monitoring invasive devices. This automation drives a critical paradigm shift from retrospective auditing to proactive surveillance, fundamentally establishing a highly accurate baseline for precision reporting with minimal burden on clinical staff (2, 7, 18, 19).

### 3.2 Predictive early warning and active intervention

Powered by AI-driven risk stratification, INIMS translate continuous surveillance into actionable insights. Clinicians can intervene at the earliest signs of transmission—even detecting localized clusters as small as two cases. While ML models have demonstrated high theoretical accuracy in predicting rapid-onset conditions like sepsis (19, 20), a crucial distinction must be made between algorithmic predictive performance and real-world clinical outcomes. Robust prospective evidence demonstrating sustained long-term mortality reductions directly attributable to AI interventions remains limited, necessitating rigorous, large-scale randomized evaluation (21).

### 3.3 Spatio-temporal management of multidrug-resistant organisms (MDROs)

Equipped with IoT spatio-temporal mapping, INIMS enable the strict, full-process management of MDROs. By precisely tracking the physical trajectories of patients, staff, and shared equipment, hospitals can help sever cross-transmission chains. Compared to manual contact tracing, intelligent identification systems drastically accelerate MDRO detection and curb secondary outbreaks through real-time digital alerts (2, 3).

### 3.4 IPC quality control and the “gift of time”

INIMS transition IPC quality control from subjective, sporadic audits to the continuous, objective tracking of hand hygiene, environmental disinfection, and waste disposal. By delegating these repetitive monitoring tasks to AI, early observational evidence suggests that hospitals can significantly alleviate administrative pressure under optimized conditions (22). When ergonomically integrated—minimizing alert fatigue and seamlessly aligning with clinical workflows—this automation returns a crucial “gift of time” to healthcare workers, preserving professional resilience and redirecting focus toward direct, empathetic patient care (4, 10, 11, 20, 23).

### 3.5 Strategic support in public health emergencies

During large-scale crises, such as the COVID-19 pandemic, INIMS function as high-speed strategic command centers. When PPE shortages and mandatory distancing disrupt routine bedside care, these systems establish a vital “digital presence.” This can help ensure uninterrupted process compliance and continuous monitoring without requiring close physical proximity to infection sources, utilizing predictive analytics to stabilize healthcare operations during systemic surges (1, 9, 24, 25).

## 4 .Current dilemmas and limitations in clinical application

Although Intelligent Nosocomial Infection Monitoring Systems (INIMS) possess transformative potential, their clinical implementation is severely constrained by structural, technical, and socio-technical friction.

### 4.1 Technological friction: interoperability, opacity, and alert fatigue

Persistent Data Silos: The primary barrier to hospital-wide INIMS deployment is the prevalence of legacy systems with proprietary, vendor-bound architectures. A lack of standardized application programming interfaces (APIs) creates “Vertical Silos,” making semantic data unification across departments cost-prohibitive (26). Consequently, global surveys reveal that less than 12% of electronic surveillance systems possess architectures stable enough for comprehensive, simultaneous hospital-wide monitoring (5).

Algorithmic Opacity and “Label Leakage”: Operating predominantly as “black boxes,” opaque AI models foster frontline distrust and introduce the risk of clinical “deskilling”—a dangerous culture of blind compliance (8). Furthermore, predicting clinical interventions (e.g., empirical vancomycin administration) rather than underlying physiological deterioration introduces “label leakage.” Such models replicate historical biases and render the AI theoretically accurate but potentially clinically limited for early warning (12, 17, 24). Furthermore, algorithm training suffers from severe deficits in dataset representativeness. Machine learning models trained exclusively on homogeneous data from well-resourced, high-income tertiary centers often lack cross-demographic validity. When directly exported to diverse populations or resource-limited settings, they risk hardcoding algorithmic bias, leading to the systemic misclassification of underrepresented patient cohorts and inadvertently exacerbating existing health inequities (8, 12, 24).

The Workload Paradox: The most pervasive implementation failure is the “workload paradox,” driven by severe dataset imbalances. Because actual infections typically account for less than 4% of hospital data, symmetrically trained algorithms often misclassify minor physiological fluctuations as threats. While techniques like the Synthetic Minority Over-sampling Technique (SMOTE) or class weighting attempt to rectify this, they pose severe overfitting risks (21, 27). Methodologically, synthetic sampling must be strictly confined to the training dataset; applying SMOTE prior to cross-validation introduces severe data leakage, leading to artificially inflated sensitivity metrics that may fail entirely during prospective deployment (8, 12). Overwhelmed by false-positive alerts, infection control specialists experience severe alert fatigue, transforming the system into an administrative burden (16, 20).

### 4.2 Clinical implementation dilemmas: systemic and ergonomic barriers

Infrastructural Deficits and Digital Inequity: The resource-intensive nature of advanced INIMS may widen the digital divide (8). Primary hospitals and community clinics often lack the broadband capacity, hardware resources, and dedicated IT staff needed for high-end IoT deployment, creating substantial disparities in infection control between resource-rich and resource-limited settings. In addition, the long-term financial burden of AI infrastructure—including software updates, cloud storage fees, and specialized IT support—threatens system sustainability. Without dedicated funding, these recurring costs may render INIMS economically unsustainable for resource-constrained hospitals (8, 10). Data Security and the “Trust Gap”: Continuous IoT data collection also raises major ethical and cybersecurity concerns. Adoption depends on narrowing the “trust gap” by ensuring that the system’s perceived clinical value outweighs concerns about cloud-based data ownership and patient privacy. Transparent data governance is therefore essential to address these barriers (9, 10, 17).

### 4.3 Overdependence, dataset shift, and medico-legal liability

Beyond infrastructural deficits, INIMS deployment risks “false reassurance,” where clinicians bypass independent judgment if the AI fails to trigger an alert. Operationally, long-term sustainability is often threatened by “dataset shift”—algorithmic degradation caused by evolving clinical baselines, as famously observed with sepsis models during the COVID-19 pandemic. Therefore, AI maintenance requires dedicated governance teams for continuous recalibration (10). Furthermore, the integration of INIMS introduces profound medico-legal vulnerabilities. Under current tort law, clinicians risk significant liability if patient injury results from following highly personalized but non-standard AI recommendations. This inadvertently incentivizes the use of AI merely as a “confirmatory tool,” while the allocation of liability among software developers, hospitals, and frontline practitioners remains legally ambiguous when opaque algorithmic errors occur (28).

### 4.4 Management and standardization dilemmas: bridging the “AI Chasm”

The Crisis of Standardization: The core of the “AI Chasm” is the absence of global standards for the construction and economic evaluation of intelligent HAI systems. Although less than 23% of ML-CDSS undergo independent cohort validation, and only 5% are evaluated in real clinical scenarios, theoretical success may be meaningless without clinical utility (5, 8). Relying solely on the Area Under the Receiver Operating Characteristic curve (AUROC) masks clinical ineffectiveness. Models must be rigorously evaluated beyond standard discrimination metrics. Incorporating probability calibration curves—ensuring predicted risks match observed frequencies—and Decision Curve Analysis (DCA) is essential to quantify the actual net clinical benefit across different alert threshold probabilities (12, 27). Deploying regional algorithms without rigorous prospective review can carry iatrogenic risks orders of magnitude higher than single human errors (11, 17).

Siloed Collaboration and Data Governance: A persistent disconnect exists between IT developers prioritizing algorithmic novelty and IPC practitioners lacking technical architecture knowledge. This fragmentation yields tools disconnected from dynamic clinical realities. Moreover, without stringent data governance, the principle of “Garbage In, Garbage Out” prevails. In developing regions, the lack of reliable microbiological data and comprehensive Electronic Health Records (EHRs) critically compromises input quality (7). Standardizing definitions—such as differentiating true infection from colonization—is an important prerequisite for INIMS to achieve ethical compliance and operational safety 5. Future Development Prospects and Optimization Strategies.

## 5 Future development prospects and optimization strategies

Bridging the “AI Chasm” requires a multi-dimensional paradigm shift, transitioning from isolated algorithmic development to holistic socio-technical integration.

### 5.1 Technological refinement: explainability and federated architecture

Future INIMS should prioritize semantic interoperability to dismantle data silos. Vendor-neutral, open-source architectures built on international standards (e.g., openEHR) enable smoother, cross-institutional data sharing via RESTful APIs. To resolve the “black box” dilemma, systems should transition toward Explainable AI (XAI). Incorporating visualization tools—such as feature-importance metrics and confidence intervals—empowers clinicians to make collaborative, rather than blindly compliant, decisions (8). Furthermore, technical evaluations should pivot from theoretical accuracy to pragmatic metrics, utilizing Positive Predictive Value (PPV) and the “Number Needed to Benefit” (NNB) to quantify the actual alert burden on clinicians (27). To expand surveillance beyond the 25.6% of device-related infections, federated learning and distributed storage (e.g., blockchain) offer secure pathways to train robust algorithms across diverse institutional datasets with minimal risk of compromising patient privacy (3, 26).

### 5.2 Clinical adaptation: “invisible” ergonomics and silent-mode validation

To prevent workflow disruption, INIMS should aim for “invisible” integration via passive monitoring, minimizing the need for active manual data entry. Abstract early warnings should be translated into actionable, visualized clinical guidelines. Crucially, hospitals should adopt standardized, phased deployments. Drawing on existing IPC frameworks (6, 20), we advocate for a recommended “Silent-Mode Validation” phase prior to official launch. During this phase, expert infection prevention teams debug real-time predictions while concealing uncalibrated alerts from frontline staff, effectively mitigating alert fatigue. Given the complexity of AI interventions, traditional patient-level randomized controlled trials (RCTs) should be supplemented with stepped-wedge trial designs to rigorously evaluate the system’s impact on operational comfort and longitudinal clinical outcomes (27).

### 5.3 Translating INIMS to low- and middle-income countries (LMICs)

To mitigate the global digital paradox, implementation in LMICs should prioritize affordability and scalability over compute-intensive architectures. Drawing inspiration from macro-level data aggregations in Iran and automated surveillance cohorts in Brazil, concrete strategies for LMICs should include deploying ‘mobile-first,’ lightweight open-source AI models via edge computing, thereby decentralizing data processing and reducing reliance on expensive cloud infrastructures. Crucially, this technological pivot should be paired with capacity-building initiatives, such as “train-the-trainer” models. Empowering local IPC nurses and IT personnel with foundational data stewardship skills can help ensure algorithms can be maintained and recalibrated independently, thereby helping to secure long-term sustainability without prohibitive vendor costs (1, 24).

### 5.4 Governance and ethics: cultivating “data solidarity”

Scaling INIMS requires unified global architecture and robust cybersecurity frameworks. Traditional privacy frameworks are fundamentally unequipped to cope with the deep ethical dilemmas of mass clinical data extraction. Global health policy must embrace ‘Data Solidarity’—a forward-looking governance framework to explicitly address the unequal distribution of digital benefits and harms.

To operate data solidarity for INIMS, there must be a structural commitment to three core pillars:

1. Facilitating high-value public data use: Given the tremendous public value generated by cross-institutional HAI predictive modeling with a low level of individual risk, such non-profit, public-health-oriented use cases must be actively facilitated, publicly funded, and provided with streamlined regulatory pathways.
2. Preventing extractive harm: Governance structures should explicitly prohibit the exploitation of hospital data for purely commercial practices, extending ethical protections beyond primary data subjects to safeguard the dignity of broader communities.
3. Cultivating a ‘data commons’: The predictive insights and structural benefits that flow from collected healthcare data must not be monopolized. They need to be managed as a common asset and returned to the public domain.

Institutionalizing these pillars will ensure that algorithmic models equitably empower all demographics and resource-limited LMICs, unequivocally preventing commercial entities from profiting at the expense of marginalized patient populations (13–15, 24).

### 5.5 .Macro-level surveillance and national biosecurity

Ultimately, INIMS should transcend individual institutional boundaries to form ubiquitous, macro-level monitoring networks, as demonstrated by Iran’s National Nosocomial Infection Surveillance (INIS) system (12). With antimicrobial resistance (AMR) projected to cause 10 million annual deaths by 2050, the deep integration of INIMS with global Antimicrobial Stewardship Programs (ASP) is important. These collaborative, automated monitoring networks have theoretical potential to serve as vital strategic assets, strengthening data-driven national biosecurity architectures. However, realizing such macro-level benefits remain highly hypothetical and will require unprecedented inter-governmental data-sharing agreements and sustained infrastructural investments to genuinely curb the global proliferation of multidrug-resistant pathogens (9, 24).

## 6 Conclusion

The transition toward Intelligent Nosocomial Infection Monitoring Systems (INIMS) is a necessary response to the escalating complexities of healthcare and antimicrobial resistance. While INIMS have proven their practical value in automating surveillance and facilitating early intervention, high theoretical algorithmic accuracy does not guarantee clinical efficacy. Bridging the “AI Chasm” requires moving beyond mere software procurement toward profound socio-technical adaptation. Sustainable implementation requires rigorous real-world model calibration, the establishment of AI governance teams to manage continuous dataset shifts, and the development of clear medico-legal frameworks. Furthermore, addressing the “workload paradox” through invisible monitoring and cultivating “data solidarity” are important to help ensure these digital tools remain scalable and equitable, particularly in resource-constrained LMICs. Ultimately, the goal of AI in infection control is not to supplant human judgment, but to return the “gift of time” to healthcare professionals, empowering them to prioritize direct, empathetic patient care.

## Data Availability

The original contributions presented in the study are included in the article/supplementary material, further inquiries can be directed to the corresponding author/s.
